# Knowledge, perceptions and experiences of trachoma among Maasai in Tanzania: Implications for prevention and control

**DOI:** 10.1371/journal.pntd.0007508

**Published:** 2019-06-24

**Authors:** Tara B. Mtuy, Matthew J. Burton, Upendo Mwingira, Jeremiah M. Ngondi, Janet Seeley, Shelley Lees

**Affiliations:** 1 Department of Global Health and Development, London School of Hygiene & Tropical Medicine, London, United Kingdom; 2 International Centre for Eye Health, London School of Hygiene & Tropical Medicine, London, United Kingdom; 3 NTD Control Programme, National Institute for Medical Research, Dar es Salaam, Tanzania; 4 Global Health Division, RTI International, Washington DC, United States of America; Emory University, UNITED STATES

## Abstract

**Background:**

The Alliance for the Global Elimination of Trachoma has set the target for eliminating trachoma as a public health problem by 2020. However, challenges remain, including socio-cultural issues. Districts in Northern Tanzania, predominantly inhabited by the Maasai ethnic group, remain endemic for trachoma. We explored socio-cultural factors that may impact the elimination of trachoma.

**Methods/Findings:**

This study was nested within a larger ethnographic study of trachoma among Maasai in Northern Tanzania. We used stratified random sampling and semi-structured interviews to examine knowledge and understanding. Interviews were conducted and recorded in Maa, by a native Maa speaking trained interviewer. Transcripts were translated into English. A framework method for a content analysis was used. There was awareness of trachoma and basic symptoms. Yet understanding of etiology and prevention was poor. Trachoma was attributed to pollen, dust, and smoke. Water was recognized as beneficial, but seen as treatment and not prevention. Traditional medicines were most often used for treating conjunctival inflammation, with the most common being a rough leaf used to scratch the inside of the eyelid until it bleeds. Knowledge of mass drug administration (MDA) was inconsistent, although many thought it helped the community, but it was perceived as only for children and the sick. Many participants reported not taking azithromycin and some had no recollection of MDA six months earlier. There was little connection between childhood infection, trichiasis and related blindness. Trichiasis was often seen as a problem of old women, and treated locally by epilation.

**Conclusion/Significance:**

Understanding indigenous knowledge may help guide control programs, tailor them to local contexts, address local beliefs and dispel misunderstandings. There is an essential need to understand the social, cultural and political context of the target community to deliver effective programs. Despite limited knowledge, the community recognized trachoma as a public health problem. Results have implications for disease control programs in other marginalized communities.

## Author summary

While global efforts to control and ultimately eliminate trachoma have been successful in many contexts, it has proven to be more challenging in many societies. Due to social, political or economic vulnerabilities, the approach to delivering global health programmes to some marginalized communities requires a more social science perspective. The Maasai, semi-nomadic pastoralists predominantly spanning the central border of Tanzania and Kenya, are one such community in which trachoma is endemic despite efforts to deliver interventions. Trachoma control interventions require community understanding of trachoma and behavior change. Furthermore, it is important to consider the community’s perspective to account for socio-cultural factors that may guide the design of effective control programmes and increase uptake of the interventions. This paper explores the knowledge and understanding of the nature of trachoma including pathology, progression of disease, risk factors, prevention and treatment among a trachoma endemic Maasai community. These findings can help guide more effective public health approaches to implementation of trachoma and other disease control interventions in Maasai communities.

## Introduction

Trachoma is the commonest infectious cause of blindness worldwide, caused by *chlamydia trachomatis* and remains a significant public health concern. This neglected tropical disease (NTD) tends to mostly impact poor and underdeveloped areas. Current estimates indicate about 182 million people live in trachoma endemic areas and is the cause of blindness or visual impairment of 1.9 million people in 42 countries [1].

The clinical features of trachoma are divided into those related to ‘active’ disease which characterizes episodes of infection and are most common in children under 10 years; and those associated with scarring. Early stages of trachoma are characterized by follicles and inflammation in the conjunctiva of the upper eyelid. Contraction of scar tissues causes eyelids to turn inward (entropion). Trichiasis occurs when eyelashes touch the eyeball. Eventually a number of factors including corneal trauma and secondary infection can lead to blindness [2]. While prevalence of active trachoma is fairly similar across sexes; women tend to have more scarring, more trichiasis and subsequently more loss of vision likely due to greater life-time exposure to infection from young children [3]. There is a dearth of information related to trachoma among marginalized ethnic groups including the Maasai of Tanzania.

The Maasai are semi-nomadic pastoralists predominantly spanning the central border of Tanzania and Kenya. The traditional lifestyle of the Maasai is changing with reduced access to land for grazing and changes in weather leading to a more semi-nomadic or even agricultural based lifestyle. Maasai are facing challenges that may negatively impact health including increased drought, poor access to major roads and education, substandard health services and on-going land disputes [4–6]. Food insecurity was found to be severe and vaccination coverage the lowest among the Maasai when compared to five other tribes in Northern Tanzania [7]. For trachoma, baseline surveys in 2006, reported trachomatous inflammation-follicular (TF) prevalence of 57.6% in children aged 1–9 years [8]. More recent studies showed the prevalence of conjunctival follicles, papillary inflammation and scarring among a cohort of children aged 6–10 years in a predominantly Maasai community in northern Tanzania, was 33.6%, 31.6% and 28.5%, respectively [9].

Risk factors for trachoma span environmental, socio-economic and behavioral factors. Risk factors for trachoma include limited access and use of water [10]; limited face washing [11–13]; poor sanitation [14, 15]; and crowding [16]. However, in marginalized communities such as the Maasai, communal living and poor economic, social and environmental conditions are a challenge to maintaining proper hygiene [17]. Control of trachoma is based on the SAFE Strategy, established by WHO in 1997 under the Alliance for the Global Elimination of Trachoma by 2020 (GET 2020). SAFE includes four public health interventions: Surgery for trachomatous trichiasis; Antibiotic treatment to eliminate the infection; Facial cleanliness promoting hygiene to reduce transmission; and Environmental change which includes management of human and animal feces, cleanliness to reduce flies, crowding and access to water [18].

SAFE has not been fully implemented in this context. Mass drug administration of azithromycin has been conducted in all high trachoma endemic Maasai districts in Tanzania in accordance with the National NTD Control Program and WHO, although impact surveys are still ongoing. Surgical camps to reduce the backlog of trichiasis cases have been ongoing in Maasai districts with the support of several international implementing partners and the National NTD Control Program. However, programs addressing facial cleanliness and environmental change components of SAFE are limited particularly in Maasai communities.

Trachoma control interventions require community understanding of trachoma and behavior change. Furthermore, it is important to consider the community’s perspective to account for socio-cultural factors that may guide the design of effective control interventions and increase uptake of the SAFE strategy. The aim of this study was to explore the knowledge and understanding of the nature of trachoma including pathology, progression of disease, risk factors, prevention and treatment among a trachoma endemic Maasai community. These findings can help guide more effective public health approaches to implementation of the SAFE strategy in Maasai communities.

## Methods

### Setting/Sampling

This study was conducted in Sinya Ward in Longido District, of Northern Tanzania. Sinya is located in the plains between Mt Kilimanjaro and Mt Meru. Sinya is comprised of three villages, Il Donyo, Leremeta, and Endonyoemali; with a total population of 4285. The community is nearly all Maasai most of whom have permanent bomas, in the village. A boma is a Maasai homestead headed by one male, consisting of houses for each of his wives and their children. A boma can range in size of 10–70 people but on average is approximately 40 people. There are a few non-Maasai, *Ormeek*, staying in Sinya for the purpose of government work in the schools and dispensaries and for trade. The main source of livelihood has been traditional livestock production in this purely pastoralist community.

Of 107 bomas in Sinya, five bomas were randomly selected in each of the three villages to achieve a sample size of 30 participants. For the purpose of this study a boma was considered a household since decisions are made by the male head of the boma. A boma also physically acts as a household in that it is a fenced enclosure of all homes or huts of the wives which are systematically placed in order of marriage. It was expected that in this traditional, isolated, Maasai community with little variation in lifestyles, 30 participants representing all three villages and different bomas would be a representative sample of the larger community. Census data was collected by the lead author for the 15 selected bomas. Each boma was visited by the lead author and together with the male head of the boma a list of all people ages 18–50 residing at the boma was documented. Using the census data, one male and one female aged 18–50 years were randomly selected to be interviewed from each of the 15 bomas. An internet-based sample builder was used to randomly select five men and women from each boma (www.randomizer.org). If the first randomly selected person was not available, the next person in the randomization list was approached to participate. As the researchers had conducted the census at each boma, most selected participants were already familiar with the researchers and a rapport had been established.

### Interviews

Semi-structured interviews were conducted from October to December 2016 with participants in Maa (Maasai language) by a native Maa speaking interviewer in a conversation-like manner. Interviews were conducted in a private setting, typically under a tree, at the participant’s home with only the participant, interviewer and principal investigator present. The interview guide consisted of socio-demographic information and open-ended questions on experiences, knowledge and understanding of the nature of trachoma including pathology, progression of disease, risk factors, experiences, treatment, prevention and blindness. Interviews were audio recorded and later transcribed and translated from Maa to English (S1 Text).

### Medicinal plant identification

Participants were asked about local treatment for trachoma and a list of plants in Maa was compiled. Two Maasai field assistants identified and photographed the plants in the field. The list of Maa plant names and photographs was used by a botanist to identify the botanical names.

### Data management and analysis

Transcription was done directly from Maa to English; some transcripts were corrected to ensure more understandable English while assuring meaning was not changed. English transcripts were entered into NVIVO 11 Software. Initial interpretation included familiarization of the data and review of reflective notes. Data were coded by lead author, TM, using the interview guide as a framework and verified by author SL. A framework method for content analysis [19] was used and descriptive findings reported. Open coding was conducted on five transcripts to confirm there were no emerging codes to be included in the analysis. Codes were grouped into themes and compared against the interview guide (S1 Table). Themes reflected the key topics from the interview guide. Using a coding framework, data were charted into a framework matrix (S2 Table). Impressions and interpretation of the framework matrix were discussed with the native speaking interviewer and coauthors. The findings reported include the key high-level codes. Constant comparative analysis was done comparing responses between genders and within bomas. Quotes presented are used to show dominant views from the interviews. Not all interviewees views are represented but rather more overarching themes included.

### Ethics statement

This study was approved by the Ethics Committees of the National Institute for Medical Research, Tanzania and the London School of Hygiene & Tropical Medicine, United Kingdom. Informed consent was obtained from all participants in Maa and a witness was present for illiterate participants. Permission to digitally record interviews was obtained from each participant. Permission from the male boma elder of the 15 selected bomas was also obtained.

## Results

### Participant characteristics

A total of 28 adults, 15 women and 13 men, from the 15 study bomas in three villages were interviewed. There were no men available for interview at two bomas due to seasonal migration in search for good pastures and business travel. Despite data saturation being reached, researchers continued to conduct interviews to assure a sense of inclusivity in the community. The participant ages ranged from 18 to 49 years. Exact age was unknown to the majority of participants as they do not maintain documentation of date of birth nor track their ages. Estimated age ranges of participants were as follows: 18–29 years (n = 10), 30–39 years (n = 9), 40–49 years (n = 9). Education level of participants were as follows: no formal education (n = 22), attended primary school (n = 5), attended secondary school (n = 1). All were conducted in a private setting, however for one interview a husband [8–1] insisted that he and his wife [8–2] be present for each other’s interview.

### What is trachoma?

The Maa term *enaoji* is a condition of the eye associated with irritation specific to the eyelid. Some mentioned white spots on the inner surface of the eyelids, possibly follicles. The most commonly reported symptoms of *enaoji* described included discharge (sometimes described as heavy and yellow), swollen eyelids, redness and itching. Some described pain, light sensitivity, inability to open the eye and an overall ill feeling throughout the body. Regardless of the associated symptoms, *enaoji* was most often explained as being specific to the eyelids.

*“… when they start to get the disease*, *there are those who get discharge from their eyes and spots on top of their [everted] eyes [lids]…”*[03–2, female in her 30’s]

*“It happens to the person when he is young when his eyes get infection and become red*. *It leads eyelids to swell due to spots and after those spots disappears is when trachoma has gone away*.*”* [6–2, female in her early 20’s]

*“It happens when people’s eyes get infection and look red*. *And then eyelids get infection which causes spots on eyelids which cause itching and a patient is forced to rub due to that*. *So*, *this is my understanding on this*.*”* [13–1, 20 year old male]

It was often reported to affect young children and some said it occurs within a few days after birth.

*“It happens when the eyelids of the child swell and in most cases children get infections to their eyes and especially young children of age about one*, *two or three are most of the cases with this problem*.*”*[02–1, 18 year old male]

*“It starts when children have discharge*, *then they get swollen [eyelids] and people say they have enaoji that’s why they are swelling*.*”* [01–2, female in her 40’s]

*“You may find a child’s eye [lids] get swollen and discharges*.*”* [05–2, female in her 40’s and a CDD in 2016]

Some described other symptoms which were not necessarily related to trachoma nor to the local interpretation of *enaoji*.

*“They can’t even open their eyes in sunny places until they get inside where it is dark*.*”* [13–2, female in her late 40’s]

*“After feeling sickness in the whole body a person feels his/her eyes are not okay*.*”* [2–1, 18 year old male]

Participants were all aware of a condition in which eye lashes turn inward and touch the eye ball, trichiasis, although they have no Maa term for it. Trichiasis was considered a normal condition that occurs with age particularly in women. None of the participants were able to link this with childhood eye infections or *enaoji* although after some probing they agreed such a link is plausible.

### Causes of trachoma and prevention of trachoma

When asked about what causes trachoma, many attributed it to pollen, dust, smoke and climate conditions that seem to vary with the year. Some mentioned “year of the eyes” in which some years there are a lot of eye problems compared to other years. Some participants attributed *enaoji* as a result of magic or a curse being inflicted by someone.

*“We believe it happens because other people applied magic power or the patient is cursed*. *So*, *we believe maybe the patient did wrong things and was cursed by his/her fellows*. *We believe that eyes and legs are among the most important body parts and life of the human are in his/her eyes and legs*.*”* [03–1, 45 year old male]

A link to flies was described by many participants but the mechanism was not clear. Some thought the flies had to bite the eye or a part of the body or the fly gets into the nose and goes up to the eyes causing *enaoji*. Others said babies were born with dead flies in their eyes which caused eye problems. Some discussed that when it rains and when there is more milk around during calving season, there are more flies and that was when more children were getting *enaoji*. Only one participant mentioned bacteria and flies as a vector. He was in secondary school, and had the highest level of education of all participants interviewed:

*“This is a disease which is caused by flies especially children when flies fall on eyes of children and they do carry bacteria from one person to another or one place to another*. *And those bacteria make settlements in the chambers of eyelids which later cause trachoma*.*”* [13–1, 20 year old male]

Methods to prevent infection were not mentioned by any participants. When asked if facial cleanliness can prevent trachoma, only a few respondents said that “it helps” but they were unable to elaborate more. Many described it as a treatment for yellow discharge, pain or irritation rather than for prevention. Although many reported being given information on cleanliness at clinics, most were unsure of the links between a clean environment and preventing trachoma and hence not convinced to follow the advice. Many said that hospitals help prevent disease or “only God can help”.

*“It [water] cannot help because all these years water is there but the disease is still a problem*. *God can only help and prevent*.*”* [08–1, 49 year old male]

### Treatment

Most participants said western medicine is better than local medicines or they are equally effective. Collecting local medicines from trees, plants and shrubs is part of Maasai women’s daily activities. Women prepare tea for their family from local medicines each morning depending on several factors including the weather, activities family members are involved in (ie, grazing, setting out on a long journey), food availability and current illnesses. Some said they go to hospitals if the local medicines do not help. A few participants believed that local medicines are better.

*“Somehow these western medicines are better because they have directives about how to use*. *These local medicines are good but due to lack of what quantity should be used sometimes it’s a challenge*. *Also*, *they are not filtered like western medicine so sometimes you may put them into your eyes with contaminations which might cause more problems*.*”* [02–2, 20 year old female]

Most participants, however, reported using local medicines or veterinary medicines (such as penicillin and oxytetracyline) for eye problems, mostly because of poor access to western treatments. These included roots, leaves and bark of various trees and plants (Table 1), or other household products (Table 2).

**Table 1:** Plant medicines used for treatment of *enaoji* (eyelid irritation).

Botanical Name	Maa Name	Description of Use
*Licium* spp Solanaceae	engokii	Boil the root with water, let it cool down and put the liquid direct into the eye.
*Cyphostemma* spp, Vitaceae	olorondo	Press the leaves, squeeze out liquid & put the liquid direct into the eye. Some mix it with sugar, salt and water.
*Grewia bicolor*	esiteti	The leaves of the plant are used to scratch the inside of the eyelid. They scratch until blood flows from the eyelid.
*Aloe volkensii* Engl.	osukuroi	Drop the liquid direct into the eye.
*Cordia monoica*	eseki	(1) Warm the wood of the plant and use it to put marks on the face of the child with belief that it will prevent eye problems. (2) Use leaves to scratch eyelids.
*Solanum incanum* L.	ndulele	Boil the root with water, let it cool down and put the liquid direct into the eye.
*Solanum taitense* Vatke	endemelua	Boil with water, let it cool down and put the liquid direct into the eye or use to wash the face.
*Olea europaea* subsp. c*uspidata*	oloirien	Warm the wood of the plant and use it to put marks above the eyelid or on the face of the child with the belief that it will prevent eye problems.
*Cammiphora swynnertonii*	oltemwai	Take the liquid/sap from the wood and put direct into the eye.
*Acacia nubica* Benth.	oldepe	Remove bark and green from a branch, cut into small pieces and boil in small amount of water (make it more concentrated). Let it cool and put the liquid direct into the eye.

**Table 2:** Other local treatments for *enaoji* (eyelid irritation).

Treatment	Description of Use
tobacco	Pour water into the side of a cigarette with the tobacco until it mixes with the tobacco and come out on the filter end. Then press the filter to drop the tobacco water mixture into the eye.
tea	Tea leaves are boiled with water, strained and warm tea is used to wash the face and eyes.
salt	Mixed with water and put direct into the eye.
sugar	Mixed with water and put direct into the eye.
milk fat from cow + tobacco	Mixed together and put direct into the eye after scratching the eyelid.
oxytetracycline (veterinary)	Put direct into the eye.
soda ash + sugar	Mixed with water and put direct into the eye.
blood + animal fat	Drink.
goat milk + soda ash	Put direct into the eye.
blood of black sheep	Put direct into the eye.
milk fat from cow	Put direct into the eye after scratching the eyelid with plant leaves.
razor blade	Used to put a mark on top outer part of eyelids so blood comes out.

**Fig 1:**
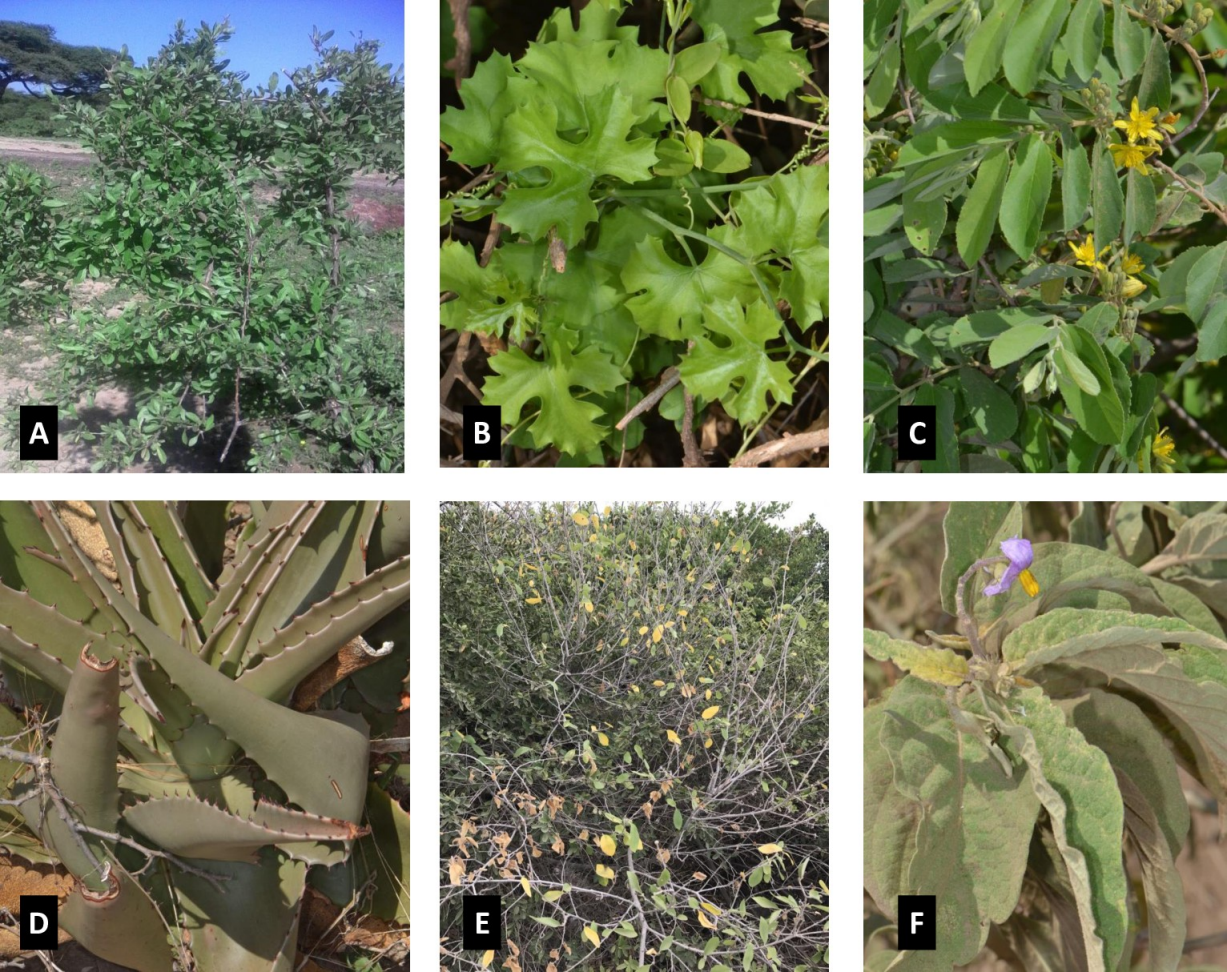
Photo of plant leaves commonly used for *enaoji* (eyelid irritation) treatment (botanical name/ Maa name). (A) *Licium* spp Solanaceae/ engokii (B) *Cyphostemma* spp, Vitaceae/ olorondo (C) *Grewia bicolor/* esiteti (D) *Aloe volkensii* Engl./ osukuroi (E) *Cordia monoica /* eseki (F) *Solanum incanum* L./ ndulele.

In most cases eye treatment for children is administered by women and the most common local treatments for *enaoji* are brewing medicines for eye drops, direct application of liquid from leaves or scratching the eyelids with a rough leaf. For the later practice, women take the leaf of the plant, *Grewia bicolor*, (Fig 1) and rub the inside of the inverted eyelid until it bleeds.

*“Instead of going to hospital*, *I first go look for a local medicine known as olorondo*. *After using that local medicine*, *if they eye is still not healed*, *I go find another local medicine from root of a plant*, *engokii*. *And if situation continues to be worse we take a leaf of a certain plant*, *esiteti and find a person to help to scratch inside of eyelids because we believe that enaoji is the problem and treatment for enaoji is scratching*.*”* [02–2, 20 year old female]

*“I have my young child who a few days ago they scratched [his eyelids] with a plant leaf due to trachoma*. *He had that problem since few days from when he was born and after scratching now he is ok*.*”* [08–2, female in her 30’s]

Although trichiasis was not considered a health condition that can be treated with surgery, most described the use a U-shaped iron, *olputet*, for epilation of eye lashes. Only one participant talked about surgery an old woman in her boma previously had, however on probing it seemed to not be related to trichiasis.

*“You may find an old person whose eyelashes turn inward and scratch the eyeball*. *And you can hear a person asking for help from another person to take off that eyelash*. *After using olputet*, *then the person feels happy*.*”* [04–1, 32 year old male]

Mass drug administration of azithromycin was conducted in Sinya in 2015 and 2016. Most participants recalled the MDA although a few were completely unaware of the program. Most reported that the drugs were probably effective because after the distribution there were fewer eye infections in the community although most were unsure how it helps. Some reported side effects either they or others had including diarrhoea, vomiting and dizziness. Some also reported uncertainty and lack of trust in the drugs:

*“We just use them and the story ends there*. *We are not sure if they gave us drugs to stop us from getting pregnant*. *We had the same drugs given to us in 2015 and we used them too*. *And to the adult like me we took two tablets*. *And we feared about those drugs that maybe they are given to us purposely to stop pregnancy*.*”* [10–2, 38 year old female]

*“I don’t know [how MDA helps trachoma] because people are ignorant so some took drugs but they didn’t use them due to fear and lack of knowledge about what these drugs are for*.*”* [12–2, 38 year old female]

### Blindness

When asked what causes blindness, most responses were that it is a result of aging. Some other causes mentioned included trichiasis, untreated eye diseases and God. If someone becomes blind at a young age, it was attributed to someone using witchcraft on them. A few women and a man talked about it as a curse for not paying traditional birth attendants after the birth of their child. Despite the traditional beliefs on causes, it did not negatively impact on how they treated the blind person.

All participants remarked that blindness is a serious disability for the individual as they are unable to attend to their daily activities without the assistance of others. They all said it was a significant burden on others in the boma in regards to taking on their responsibilities at the boma including cooking and cleaning and assisting them to the toilet and other basic needs. The economic burden was also mentioned.

*“The burden is big*. *Like the one [blind mama] we have at this boma…because she depends on other women in the boma to go look for firewood for her*, *collect water in the dam and even to repair her house*. *Other women are responsible to do that*.*”* [15–2, 22 year old female]

*“The burden is very big*. *I’m wondering about the situation my son is facing*. *For example*, *when I go outside of his mother’s house to stay with other men he follows me like you see here now*. *And during the night when he wants to go back to his mother’s house he asks me who will take him back because he can’t go alone*. *So*, *it is a big problem to the young boy like this to lose vision at this age*. *It is also a burden to us who are supposed to take care of him*. *Like when I hear him crying on the other side of the boma*, *I always get scared because I think he got into thorns*.*”* [03–1, 45 year old male]

Although blindness was considered a burden on the community, all participants discussed supporting blind people in their daily activities. Some family members, including children, were appointed as caretakers.

There was little difference in responses between men and women. Women tended to be more descriptive in the symptoms of *enaoji* as well as the practice of scratching eyelids for treatment. Women were more likely to discuss side effects and rumours of MDA. Analysis of the data between the man and woman within a boma, showed similar responses. This consistency of responses within a boma indicates sharing of knowledge and perceptions around health between men and women.

## Discussion

The Maasai have a strong cultural identity and maintain a traditional lifestyle while having limited access to health services. Despite a high prevalence of trachoma in Maasai communities, their experiences and knowledge of trachoma have not been explored. A barrier to effective control of NTDs is lack of knowledge of the disease in the community. Despite the complexity of understanding a community and its response to disease, sharing knowledge is important for engaging the community to perhaps change perceptions and behaviours. Yet, information based interventions alone are often not effective for behavior change [20, 21]. It has been shown that knowledge alone for trachoma control often does not change behaviours in the short term [20, 21]. Programs should consider multiple techniques to engage participants at behavioral, social, sensory and cognitive levels [22]; leverage non-health-related motives, and/or improve poor habits (e.g., via nudges, and reminders) [23] for sustainable behavior change. Yet underlying these components is an essential need to understand the social, cultural and political context of the target community to deliver effective programs.

There is a danger in approaching health education from a biomedical perspective and not incorporating local understanding, beliefs around illness and traditional healing [24]. In this study, only one participant had secondary education and he had a more accurate understanding of trachoma than any of the other participants. Haasnoot [25] found education of Maasai children was significantly associated with knowledge of TB in terms of awareness and understanding its aetiology compared to adult Maasai with no primary education. School-based interventions may be effective in delivering public health messages to the broader community in areas with low education. Furthermore, control measures are often not compatible with a community’s traditional beliefs, practices and understanding of the aetiology, transmission, prevention and treatment. Often the complexities of how a society interprets a disease and the cultural beliefs around prevention are reduced to a crude description of that society. Coast [26] describes this in relation to use of condoms for HIV prevention and the belief among Maasai men and women that condoms waste semen and semen exchange is highly valued by both sexes. Yet NGOs and health workers blame poor condom use on a sexually deviant society rather than the complexities of Maasai sexuality.

This study has shown that the Maasai community is aware of basic clinical symptoms of childhood infections related to eye conditions, particularly related to the eyelids, and that they have a Maa term for it, *enaoji*. However, many of these symptoms are not specific to trachoma. Therefore, *enaoji* may not always be attributable to trachoma, but is understood in terms of an inflammation of the eyelids. Maasai have a traditional practice of using a hot iron to put circular scars on the cheek under the eyes of children. Although today this practice is mostly done for aesthetic reasons, it originated as a means of protecting the individual from *enaoji*. This suggests that *enaoji* is identified by Maasai as a serious problem, despite their poor clinical knowledge. For example, their descriptions of eye infections in infants occurring from birth refers to ophthalmia neonatorum, and is usually caused by bacterial infections acquired during delivery, *Chlamydia trachomatis* and *Neisseria gonorrhoeae*. Participants in this study were very aware of a condition in which the eyelashes turn in and scratch the eyes, trichiasis, although they considered it normal and part of aging. All were unaware of a link between childhood infections and trichiasis, a factor that may limit uptake of interventions. This is consistent with reports from Guinea Bissau [27] and the Gambia [28]. Whilst, there was a lack of understanding of the aetiology of trachoma many recognized an association between presence of flies and increased infection. Rather than a clinical understanding, this study has revealed that for the Maasai trachoma is attributed to either environmental conditions and/or related to spiritual beliefs (see also Sindiga 1995). In line with our findings other studies looking at other NTDs in other low and middle-income countries similarly found that beliefs on disease aetiology were environmental or spiritually based [29–33]. For example, a study in the Gambia revealed bad air was believed to cause trichiasis [naturalistic cause] and bad air affects eyes because ‘enemies’ in the village wish to afflict them [personalistic] [28].

Our findings on poor understanding of cleanliness and disease prevention (such as face washing and home environment) are corroborated by a study in Guinea Bissau which found only 25% in the rural setting believed there were benefits in good hygiene and were unable to discuss any preventive measures [27]. However, in another study in the Gambia women had the knowledge and understanding of a link between personal hygiene and disease prevention although activities related to cleanliness ranked of lower priority in their daily activities [28]. Instead, the Maasai approach to disease prevention is use of plants including the daily consumption of tea from herbs, barks and roots for prevention of various conditions or *orpul*, the commonly practiced healing retreat to maintain health and vigour [34–36]. SAFE strategies should be tailored to the local context to address local beliefs and help dispel misunderstandings [37].

In relation to treatment, although the Maasai trust western medicine, they primarily use local medicines to treat eye conditions. This may possibly be due to isolation from centers of development [38] and a strong cultural identity [39]. In this area of 223 square kilometres there are only two health facilities. Some residents walk up to 15 km to a clinic often to find no doctor on duty or medicines unavailable. A study conducted with the Maasai of Southern Kenya, found 73% of participants indicated traditional medicine was their preferred form of treatment. However traditional medicine is not the sole form of medical treatment as 98% at times seek medical attention from local dispensaries and clinics [34]. Reluctance to attend a clinic for care may also be linked to how patients are treated when they go to health facilities [40]. The clinic staff do not speak Maa and patients have reported being yelled at for presenting to the clinic late. Health services that demonstrate a lack of consideration for cultural beliefs risk being rejected by the community [40].

Despite the community having numerous outreach programs for trichiasis surgery none of the participants were aware what it was for, indicating a lack of education and sensitization from the surgical camps. This is not surprising considering the communities’ perception of trichiasis as a natural part of aging and not connecting trichiasis to childhood eye infections. Lack of awareness of trichiasis surgery was also reported in trachoma endemic communities in the Gambia and central Tanzania [41, 42]. A few community members have had cataract surgery and there was more understanding about surgery to improve vision than surgery to protect vision through correcting in turned eyelashes.

While participants were aware of MDA, all were unsure of how it helps and complained of side effects. Effectiveness of an MDA program often depends on community mobilization and sensitization. This includes addressing possible side effects and ways to prevent them including not taking the drug on an empty stomach. Lack of sensitization around MDA was associated with poor uptake of azithromycin in a study in Kilimanjaro Region of Tanzania in which 56% of respondents reported being satisfied with the amount of information they received prior to MDA and 32% reported side effects including diarrhoea, stomach ache and nausea [43]. In our study, a few women said they heard the drug can cause infertility. This is similar to studies in Ethiopia and Kenya in which women perceived azithromycin as a form of family planning [44, 45]. The origin of rumors can be complex. On one hand, they can arise due to misinformation, whilst on the other hand rumors may have a historical or political origin or result from cultural beliefs [24]. The act of passing on rumors may not be related to whether the person passing it on believes the rumor [46] but rather a response to someone’s mistrust or uncertainty of something.

Despite men and women having differential risk and experiences with trachoma, there was little difference in the responses between men and women. This is likely due to the continual exchange of knowledge and experiences of health within the Maasai [47] that may contribute to the little differences in responses within bomas. Women tended to have more descriptive responses likely due to more direct experience caring for children with active infection and being responsible for health of the children [48]. Although men are the ultimate decision makers regarding the use of resources for household members to seek health care, they respect women’s judgements regarding health issues and needs to seek health care.

This study had some limitations. Interviews were conducted in the local Maasai language, Maa, by an experienced research assistant. Despite adequate training on trachoma and interview techniques, probing was sometimes not adequate. The lead author was present for all interviews but due to limited understanding of the language was not always able to assist in probing when necessary. The lead researcher, a non-Maasai or *ormeek*, was aware of potential misunderstandings of her position given that the Maasai encounters with non-Maasai tend to be with NGO, government or clinical personnel. To gain trust the lead author lived in the community for sixteen months and spent significant amount of time engaging local leaders in the research. Further to this, Sinya may not be representative of all Maasai communities in Tanzania. This community was chosen as it is traditional and remote in terms of its exposure to bigger towns and cities. Therefore, access to health facilities, health programs and education are likely more limited in Sinya compared to many other Maasai communities. Despite this, it is important to note that the communal nature of Maasai culture is still very traditional and consistent across different Maasai districts and economic levels. This study focused on knowledge and experiences which alone will not change behaviour [20]. Therefore, it is important to consider the political, social and economic context in which the lives of the people are situated. This study did not explore this context in depth. This study was nested within a larger ethnographic research project investigating the Maasai response to MDA for trachoma from a political economy perspective.

### Conclusion

This is the first study to examine perceptions and experiences of trachoma among the Maasai in Tanzania. Additionally, this was the first study to identify a local treatment used in treatment of swollen eyelids among the Maasai. In particular the commonly practiced scratching of eyelids with a rough leaf has not been documented. Children’s eyelids are scratched until they bleed. This may potentially lead to secondary scarring related to this local practice. Further studies are needed to explore the effects of local treatment on scarring and progression of disease.

This study found prevention of blindness is important to Maasai. They discussed the social and economic burdens of blind people in the community. Possibly if health education in the community included the connections of childhood infections, trichiasis, and blindness, the community would place greater value on the information to prevent this disability.

It is important to understand the indigenous knowledge of disease to guide effective control programs. Therefore, further ethnographic research with an in-depth focus on this communities’ beliefs, practices and relationships with health care is needed. The National NTD Control Programme has put resources into behaviour change interventions for trachoma control. These findings will provide further understanding of the community to tailor interventions more appropriate for Maasai. Additional research is needed to further explore the effect of a multi-level behaviour change intervention on sustained behaviour change for improved F and E practices among marginalized communities such as the Maasai. While this study focused on the Maasai in Tanzania, the results may contribute to the broader knowledge base and approach to improving control programs for other marginalized communities.

## Supporting information

S1 Text Interview topic guide. (PDF)

S1 Table Coding framework. (PDF)

S2 Table Summary of results by coding framework. (PDF)
