# Supplementary material for: Knowledge, perceptions and experiences of trachoma among Maasai in Tanzania: Implications for prevention and control
Source: PLoS Negl Trop Dis. 2019 Jun 24;13(6):e0007508. doi: 10.1371/journal.pntd.0007508 (PMC6611635; doi:10.1371/journal.pntd.0007508)
Supplement: S1 Text — (PDF) [file pntd.0007508.s001.pdf]

Interview Topic Guide

General Information:
- Date
- Location
- Name of interviewer
- Start time
- End time

Introduction:
- Aim of interview
- How long it will take
- What happens to the data
- Confidentiality
- Consent process

1. Demographic information
   a. Gender
   b. Age
   c. Village/sub village
2. Have you ever heard of trachoma? What do you know about it? *(or show pics of people with trachomatis disease to elicit information)*
3. What are some of the symptoms people with trachoma experience?
4. Who is at risk for getting this eye disease?
5. Do you know any children with discharge from the eye? Can you describe the eye problem or eye disease?
6. How does someone get this disease? What causes this?
7. Do you know of any ways to prevent the disease? How? *(probe if know about facial cleanliness and environmental issues)*
8. Do you recall when drug distributors have come to the village to give you a drug for trachoma? How does this drug help trachoma?
9. Treatment
   a. What do you do when you or your family members have eye problems?
   b. What treatments are you aware of other people using
   c. Perceptions of western treatment vs local medicine
10. Have you ever come across a disease where the eyelashes turn inwards and scratch the eyeball?
11. What is the treatment for trichiasis?
    a. *Can explain this is what people carry 'olputetu' for.*
12. How is that children have the infection but 'eyelash trachoma' /trichiasis (what leads to being blind) occurs as adults?
13. Beliefs about blindness and its causes.
    a. What causes blindness?
    b. How is blindness perceived. Stigma.
    c. What is it like for a blind person to be cared for/ burden on others in the boma.
