# Supplementary material for: Knowledge, perceptions and experiences of trachoma among Maasai in Tanzania: Implications for prevention and control
Source: PLoS Negl Trop Dis. 2019 Jun 24;13(6):e0007508. doi: 10.1371/journal.pntd.0007508 (PMC6611635; doi:10.1371/journal.pntd.0007508)
Supplement: S1 Table — (PDF) [file pntd.0007508.s002.pdf]

Coding Framework	
**Theme**	**Codes**
**What is trachoma?**	symptoms
	infected populations
	trichiasis
**Causes of trachoma**	environmental
	supernatural causes
	flies
**Prevention**	cleanliness
	western medicine
	God
**Treatment**	health seeking behavior
	local treatment
	western treatment
	vetinary medicine
	God
	decision making
	MDA
	epilation for trichiasis
**Blindness**	causes
	burden
	support systems
