# Supplementary material for: Knowledge, perceptions and experiences of trachoma among Maasai in Tanzania: Implications for prevention and control
Source: PLoS Negl Trop Dis. 2019 Jun 24;13(6):e0007508. doi: 10.1371/journal.pntd.0007508 (PMC6611635; doi:10.1371/journal.pntd.0007508)
Supplement: S2 Table — (PDF) [file pntd.0007508.s003.pdf]

Summary of Coding Framework								
		Themes						
boma	gender	what is trachoma	symptoms	causes of trachoma	prevention	treatment	MDA	blindness
01	male		pain	pollen	wash eyes, clean whole body, avoid flies	God, veterinary meds, local tx	helped	burden, support, local tx, caused by TT
	female	children, irritation	discharge, crying, red	other diseases	*(unsure)*	local tx	helped	old age, curse, burden, support
02	male	kids, eyelid irritation	swelling, whole body sick, eyes not well	poor sanitation, flies	hospital and medicine	local tx	helped	old age, curse, burden, varies by year
	female	eyelids	discharge, swelling, pain, white layer on cornea	sanitation, dust	medicine, keep house clean	local tx	helped children	old age, curse, burden,
03	male	everyone at risk, TT	discharge, yellow fluid	magic/curse	*(unsure)*	western meds, veterinary meds, hospital	didn't see difference	burden, unsure cause
	female	children, eyelids, lead to blindness	spots, swollen, red, can't open eye	born with it, flies, pollen	*(unsure)*	local tx	help, side effects, not sure what they for, take b/c gov't says so	unsure cause, burden, support
04	male	born with it, TT	light sensativity	dust, pollen, eye injury	hospital, medicine, clean environment, wash face	western meds	helped, side effects	from disease
	female	linked with seasons, TT	red, swollen	sanitaiton, pollen, flies, God	*(unsure)*	western meds, local tx	helped	TT, burden
05	female	children, TT	swelling, discharge, pain	seasonal	*(unsure)*		helped	children and old, burden
06	male	born with it, TT	light sensativity, discharge	smoke, sanitation	hospital, medicine	vetinary meds, local tx, western meds	was traveling	God causes, old age, western med helps burden
	female	children, eyelids	swelling, spots on eyelid, yellow discharge	sanitation	*(unsure)*	local tx	*(unsure)*	caused by infection (children & old), burden
07	male	sick eyes	discharge, itchy, swelling	pollen, dust	*(unsure)*	local tx	*(unsure)*	caused by western med, local tx helps
	female	foreign body in eye, TT	dicharge, swelling	pollen, dust	water	western meds	helped	caused by God and disease, burden
08	male	unsure, women know	swelling, red, itchy	weather	God	local tx	helped	old age
	female	TT, born with it	itchy, red, discharge, eyes closed in morning	pollen, whole body is sick	medicine	local tx, western meds	helped	cause by scratching, TT
09	male	TT, from scratching	yellow discharge	flies- infection, rain-milk-flies	no flies	local tx, western meds	helped, wasn't around	eye disease, burden
	female	*(unsure)*	discharge	*(unsure)*	*(unsure)*	local tx	unsure they helped	unsure of cause, burden
10	male	TT, from sick eyes	yellow discharge, red, swelling	dust, weather, flies	wash face	local tx, western meds	helped	God causes, burden, young and old
	female	*(unsure)*	red, yellow discharge, swelling	*(unsure)*	wash eyes	God, local tx	side effects, contraceptive	old age, women, disease, burden
11	male	born with it	irritation, discharge	eye injury, God, born with dead flies in eyes	God	local tx, western meds	didn't take	old age, disease, burden, support
	female	poor vision	headache, pain in eye	God, flies	God	western meds	helped	old age, disease, burden, support
12	male	can't open eye	swelling, red, light sensitivity	dust	*(unsure)*	local tx	can't recall	disease, burden
	female	*(unsure)*	dicharge, can't open eyes	climate	*(unsure)*	western meds		disease, can be treated, burden
13	male	infection	red eyelids, itching, yellow discharge	flies, bacteria, dust, flies	brand rings on cheeks, washing	local tx	didn't take, fear	caused by smoke, burden
	female	leads to blindness	infection, swelling, light senstativity	flies	*(unsure)*	western meds, veterinary meds, hospital	didn't help	disease, burden, support
14	female	*(unsure)*	discharge	God, dead flies	wash eyes	local tx, western meds	side effects	happens in children, burden
15	female	*(unsure)*	light sensativity, discharge, tearing	dust	*(unsure)*	local tx, western meds	can't recall	burden
